# Diagnostic pathways and management in women with pregnancy-associated breast cancer (PABC): no evidence of treatment delays following a first healthcare contact

**DOI:** 10.1007/s10549-018-05083-x

**Published:** 2018-12-14

**Authors:** Anna L. V. Johansson, Caroline E. Weibull, Irma Fredriksson, Mats Lambe

**Affiliations:** 1 Department of Medical Epidemiology and Biostatistics, Karolinska Institutet, Stockholm, Sweden; 2 Cancer Registry of Norway, Oslo, Norway; 3 Department of Molecular Medicine and Surgery, Karolinska Institutet, Stockholm, Sweden; 4 Department of Breast and Endocrine Surgery, Karolinska University Hospital Solna, Stockholm, Sweden; 5 Regional Cancer Centre Uppsala-Örebro, Uppsala, Sweden

**Keywords:** Pregnancy, Breast cancer, Symptoms, Diagnostics, Diagnosis, Treatment, Clinical management, Delay, Postpartum, Time-since-birth

## Abstract

**Background:**

Women with pregnancy-associated breast cancer (PABC), i.e. diagnosed during or within 2 years of pregnancy, have a poor prognosis. We compared symptoms, diagnostics, treatments, and waiting times from first symptoms to treatment initiation in women diagnosed with PABC and non-PABC.

**Materials and methods:**

Women diagnosed with PABC and non-PABC at ages 15–44 were identified in Swedish healthcare registers. Chart information was retrieved for 546 women (273 PABC cases and 273 age- and hospital-matched non-PABC controls) treated at 11 hospitals across Sweden between 1992 and 2009. Distributions of symptoms, diagnostics and treatments were compared. Median waiting times from initial symptoms to start of treatment, and time periods within, were estimated from Kaplan–Meier curves.

**Results:**

Initial symptoms in women with PABC and non-PABC were similar. Women with PABC more often underwent biopsy and ultrasound than mammography at initial examination. Compared to non-PABC, rates of mastectomy and axillary clearance were higher in women with PABC, while endocrine treatment was less common. The time from symptoms to first healthcare contact was non-significantly longer in women diagnosed during or within 6 months of pregnancy. Waiting times from contact with healthcare to diagnosis and treatment were shorter or similar in women with PABC compared to women with non-PABC.

**Conclusions:**

These findings do not support the notion that diagnostic and treatment delays following a first healthcare contact are more common in women diagnosed with breast cancer during or shortly after pregnancy. However, there was some evidence of delays in seeking healthcare among pregnant and lactating women.

**Electronic supplementary material:**

The online version of this article (10.1007/s10549-018-05083-x) contains supplementary material, which is available to authorized users.

## Introduction

Pregnancy-associated breast cancer (PABC) is commonly defined as a breast malignancy diagnosed during pregnancy or within 1 or 2 years of delivery. While PABC is a rare condition with an estimated incidence of three per 10,000 deliveries [1, 2], women with PABC more often present with hormone-receptor negative and more advanced tumours at diagnosis, leading to a poorer prognosis compared to other young women with breast cancer [3–5].

Diagnostic and treatment delays, together with lower diagnostic and treatment intensity, have been suggested to contribute to more advanced disease and poorer outcomes in women with PABC [6–9]. While pregnancy usually is a period of intense medical observation, signs and symptoms of a malignancy may be overlooked or misinterpreted as pregnancy-related, resulting in delayed diagnosis. In particular so for pregnant and lactating women, where marked physiological changes of the breast, including increased cell turnover and glandular tissue, can mask tumour growth. Also, breast cancer management, including diagnostic procedures, during pregnancy and lactation must be adapted to minimize maternal and fetal risks, and may thus be suboptimal [10–14].

Few studies to date have in detail investigated diagnostic and treatment delays in women with PABC. Most previous studies have been based on small samples or have had no comparison groups [9, 15–21], and only a few have included information on symptoms or diagnostic intensity [15, 16, 19, 20]. Taken together, the findings of these studies indicate that diagnostic delays may be present for women with PABC, while results on treatment delays are less conclusive.

We aimed to investigate if women with PABC differ to non-PABC with respect to signs and symptoms, initial investigation and treatment. In addition, we compared waiting times from symptoms to healthcare contact, diagnosis and treatment to identify possible diagnostic and treatment delays.

## Methods

The present study was based on population-based data from Swedish healthcare registers and information retrieved from medical records. We identified a cohort of 9441 women with a first record of an invasive breast tumour (International Classification of Diseases (ICD) version 10 code = C50, Patho-Anatomical Disease code = 096 corresponding to adenocarcinoma in ICD-O2 and ICD-O3) between 1992 and 2009 at ages 15–44 years in the Swedish Cancer Register (SCR). The SCR records data on all newly diagnosed cases of cancer since 1958, including information on diagnosis date and tumour site. By use of the Swedish national registration number, uniquely assigned to all residents of Sweden, the cohort was linked to the Swedish Multi-Generation Register (MGR). The MGR includes birthdates of all Swedish residents since 1961 with a link to parents. We retrieved birthdates on all live born children to women in the study cohort, allowing classification of breast cancers as pregnancy-associated or not. PABC was defined as an invasive breast tumour diagnosed during pregnancy and up to 2 years post-delivery. A non-PABC was defined as diagnosed among nulliparous or more than 2 years since most recent pregnancy. In total, the study population encompassed 778 PABC and 8663 non-PABC women. Using information from the SCR and MGR, a family history of breast cancer was defined as having a mother or sister (first-degree relative) with breast cancer. Information on educational level was obtained from the Education Register held by Statistics Sweden.

To retrieve information on symptoms, detection, initial investigation, tumour characteristics and treatment, information from medical records was manually collected for a sample of the full study population. Eligible cases were all women diagnosed with PABC (*n* = 336) at 11 large hospitals across Sweden, selected based on size and geographical location. In these hospitals, 3061 women with non-PABC were eligible for sampling as controls (Supplemental Table 1). Control women were 1:1-matched to PABC cases by age at diagnosis (in groups 15–29, 30–34, 35–39, 40–44 years) and by hospital, and randomly selected within these matching strata. In total, we were able to retrieve medical records for 273 women with PABC and 273 non-PABC controls. The retrieval rate was 82% overall (one hospital had 9% retrieval rate, while the rate in the other ten hospitals averaged 91%).

Using a standardised protocol, two trained nurses extracted information on symptoms, detection and initial investigation from medical records. One nurse abstracted data on 262 (96%) of matched PABC/non-PABC pairs, while the second nurse abstracted information on the remaining 11 (4%) of matched pairs. Because of the nature of the project with detailed review of the medical records, it was not possible to blind the nurses for the pregnancy history of the women. Data on timing of symptoms, first healthcare contact, examination and surgery (during pregnancy, or during/after lactation) were also retrieved. Treatment information included type of surgery (none, breast conserving, mastectomy), axillary surgery (none, axillary clearance, axillary sampling, sentinel node biopsy), adjuvant treatment (combinations of radiotherapy (RT), chemotherapy (CT), and endocrine treatment (ET)). Tumour characteristics included tumour size (clinical, pathological), metastasis (local, regional, distant spread), and multifocality. Information on grade and hormone receptor status was obtained from the Swedish Breast Cancer Quality Register. For a total of 226 women (113 matched PABC/non-PABC pairs) information on date of first signs or symptoms was available, while 466 women (233 matched pairs) had information on date of first healthcare contact, diagnosis and start of treatment. Waiting times were defined as A: time from first symptom to first healthcare contact, B: time from first healthcare contact to diagnosis, C: time from diagnosis to initiation of treatment, and D: time from first healthcare contact to initiation of treatment (Supplemental Fig. 1).

### Statistical methods

Numbers and proportions of outcomes (symptoms, detection, contact, examination, tumour characteristics and treatments) were calculated for women with PABC and non-PABC, respectively. The PABC group was further divided into time-windows; women diagnosed during pregnancy, during 0–6, 6–12 and 12–24 months after delivery to reflect different periods of lactation. Outcomes by PABC and non-PABC groups were compared using paired tests accounting for the matching on age and hospital; for binary outcomes McNemar’s test was used, while for categorical outcomes Stuart-Maxwell test (a generalisation of McNemar’s test) was used [22]. These tests only include 1:1 matched pairs with complete information on both the exposure and outcome. Correlation between clinical and pathological T stage was estimated using the Pearson correlation coefficient. To assess calendar time effects, we performed a sensitivity analysis with additional posthoc matching for calendar period. This reduced the analytical sample while the results were essentially unchanged. Median waiting times in days were estimated from Kaplan–Meier curves and compared using stratified log-rank tests (stratified on matching pair). All tests were two-sided and the significance level was 5%. All analyses were performed using statistical software Stata 15.1/IC [23].

This study was approved by the Ethical Review Board in Stockholm, Sweden.

## Results

Of 273 women with PABC, 41 women were diagnosed during pregnancy (one during first trimester, ten during second trimester, and 30 during third trimester), 30 during the period 0–6 months post-delivery, 64 during 6–12 months post-delivery and 138 during 12–24 months post-delivery. Of 273 women with non-PABC, 96 occurred prior to childbearing and 177 occurred more than 2 years since latest childbirth. Women diagnosed during 0–6 months post-delivery were youngest (Table 1). Compared to women with non-PABC, women with PABC had more children and a higher education at time of diagnosis. Family history of BC did not differ between the two groups.

**Table 1:** Patient and tumour characteristics by pregnancy-association at diagnosis

	PABC *N* = 273		Non-PABC *N* = 273		PABC pregnancy *N* = 41		PABC 0–6 months *N* = 30		PABC 6–12 months *N* = 64		PABC 12–24 months *N* = 138	
	*N*	%	*N*	%	*N*	%	*N*	%	*N*	%	*N*	%
Age												
15–29	28	10.3	28	10.3	7	17.1	9	30.0	6	9.4	6	4.3
30–34	100	36.6	100	36.6	18	43.9	8	26.7	23	35.9	51	37.0
35–39	98	35.9	98	35.9	11	26.8	12	40.0	23	35.9	52	37.7
40–44	47	17.2	47	17.2	5	12.2	1	3.3	12	18.8	29	21.0
*p* value		1.00				1.00		1.00		1.00		1.00
Period												
1992–1997	71	26.0	29	10.6	7	17.1	5	16.7	23	35.9	36	26.1
1998–2003	72	26.4	68	24.9	16	39.0	8	26.7	18	28.1	30	21.7
2004–2009	130	47.6	176	64.5	18	43.9	17	56.7	23	35.9	72	52.2
*p* value		< 0.01				0.96		0.53		< 0.01		< 0.01
Parity prior to BC^a^												
Nulliparous	0	0.0	96	35.2	0	0.0	0	0.0	0	0.0	0	0.0
1 child	85	31.1	32	11.7	15	36.6	8	26.7	24	37.5	38	27.5
2 + children	188	68.9	145	53.1	26	63.4	22	73.3	40	62.5	100	72.5
*p* value		< 0.01				< 0.01		< 0.01		< 0.01		< 0.01
Family history of BC												
No	243	89.0	245	89.7	32	78.0	29	96.7	60	93.8	122	88.4
Yes	30	11.0	28	10.3	9	22.0	1	3.3	4	6.3	16	11.6
*p* value		0.79				0.13		0.32		0.37		0.71
Education level												
≤ 9 years	17	6.3	31	11.5	3	7.3	2	6.7	5	7.9	7	5.2
10–13 years	98	36.4	115	42.6	13	31.7	17	56.7	21	33.3	47	34.8
13–14 years	58	21.6	45	16.7	10	24.4	3	10.0	14	22.2	31	23.0
> 14 years	96	35.7	79	29.3	15	36.6	8	26.7	23	36.5	50	37.0
Missing	4		3									
*p* value		0.035				0.46		0.83		0.17		0.42
Tumour size, cT												
T0:0 mm	6	2.6	6	2.5	1	2.9	1	4.0	1	1.7	3	2.6
T1:1–19 mm	47	20.3	66	27.4	8	23.5	5	20.0	2	3.4	32	28.1
T2:20–49 mm	124	53.4	129	53.5	18	52.9	8	32.0	37	62.7	61	53.5
T3: ≥ 50 mm	55	23.7	40	16.6	7	20.6	11	44.0	19	32.2	18	15.8
Missing	41		32									
*p* value		0.08				0.38		0.07		0.04		0.99
Tumour size, pT												
T0:0 mm	5	2.1	7	2.7	0	0.0	1	3.6	1	1.9	3	2.4
T1:1–19 mm	87	36.0	109	42.7	12	31.6	9	32.1	14	26.4	52	42.3
T2:20–49 mm	115	47.5	105	41.2	17	44.7	11	39.3	30	56.6	57	46.3
T3: ≥ 50 mm	35	14.5	34	13.3	9	23.7	7	25.0	8	15.1	11	8.9
Missing	31		18									
*p* value		0.28				0.27		0.41		0.31		0.64
Correlation^b^ between cT and pT		0.44		0.33		0.61		0.34		0.41		0.33
Metastasis												
No	149	61.8	174	69.9	28	73.7	13	44.8	30	50.8	78	67.8
Axilla only	88	36.5	70	28.1	10	26.3	14	48.3	28	47.5	36	31.3
Distant and axilla	4	1.7	5	2.0	0	0.0	2	6.9	1	1.7	1	0.9
Missing	32		24									
*p* value		0.11				0.78		< 0.01		0.23		0.21
Histological type												
Ductal^c^	186	79.1	182	79.5	28	75.7	23	82.1	43	76.8	92	80.7
Lobular	11	4.7	20	8.7	2	5.4	1	3.6	1	1.8	7	6.1
Medullar	4	1.7	3	1.3	1	2.7	0	0.0	1	1.8	2	1.8
Other	34	14.5	24	10.5	6	16.2	4	14.3	11	19.6	13	11.4
Missing	38		44									
*p* value		0.39				0.37		1.00		0.31		0.27
Grade												
I	2	1.4	11	6.5	1	4.2	1	5.3	0	0.0	0	0.0
II	29	20.6	57	33.9	2	8.3	3	15.8	4	16.0	20	27.4
III	110	78.0	100	59.5	21	87.5	15	78.9	21	84.0	53	72.6
Missing	132		105									
*p* value		0.056				0.37		0.56		0.22		0.13
ER												
Neg	102	47.9	81	34.5	21	63.6	14	63.6	23	47.9	44	40.0
Pos	111	52.1	154	65.5	12	36.4	8	36.4	25	52.1	66	60.0
Missing	60		38									
*p* value		0.011				0.025		0.10		0.17		0.51
PR												
Neg	121	57.1	103	44.6	20	60.6	18	81.8	28	60.9	55	49.5
Pos	91	42.9	128	55.4	13	39.4	4	18.2	18	39.1	56	50.5
Missing	61		42									
*p* value		0.021				0.025		0.10		0.21		0.56
Multifocality												
No	226	88.3	228	87.4	36	90.0	27	93.1	52	88.1	111	86.7
Yes	30	11.7	33	12.6	4	10.0	2	6.9	7	11.9	17	13.3
Missing	17		12									
*p* value		0.89				0.48		0.03		0.74		0.05

*p* values from paired Stuart–Maxwell test (Mc Nemar’s test) including only matched pairs with complete information

^a^Parity includes index birth for women with PABC during pregnancy

^b^Pearson correlation coefficient

^c^Ductal corresponds to ductal with/without lobular or other component

Compared to women with non-PABC, women diagnosed during pregnancy and up to 6 months of post-delivery tended to have larger tumours (pT3: 23.7% (pregnant), 25.0% (0–6 months), both non-significant), while spread to lymph nodes was more common in women diagnosed 0–6 months post-delivery (axilla alone: 48.3%, axilla and distant metastasis: 6.9%, significant). The highest correlation between clinical and pathological tumour size was observed in pregnant women compared to all other groups (Pearson correlation coefficient 0.61 (pregnancy), 0.34 (0–6 months), 0.41 (6–12 months) and 0.33 (12–24 months)). There was a tendency for high-grade tumours to be more common in PABC and especially when diagnosed during pregnancy (grade III: 87.5% (pregnancy), 78.9% (0–6 months), 84.0% (6–12 months), and 72.6% (12–24 months), non-significant). Hormone receptor negative tumours were more common in women with PABC, being most pronounced when diagnosed during pregnancy. There was no difference in histological type or presence of multifocality between the two groups.

### Signs, symptoms and initial investigation

Nearly all women, regardless of PABC status, reported a lump as their first, main symptom of which the vast majority detected the lump themselves (Table 2). There was no difference in number of symptoms between women with PABC and non-PABC. Only a few women were diagnosed at mammography screening (this is mainly an unscreened population), and none of the screen-detected women were diagnosed during pregnancy or within 12 months of delivery. A higher proportion (non-significant) of women diagnosed during or within 6 months of pregnancy had a consultation with a breast specialist at first healthcare contact, compared to women diagnosed further away from delivery. Also, mammography at initial examination was less commonly performed in women diagnosed during pregnancy and within 6 months of delivery [pregnancy: 69.2% vs. 92.3% in matched controls (*p* value < 0.01), 0–6 months: 82.1% vs. 96.4% (p-value = 0.10)]. Instead, this group more often underwent ultrasound and biopsy. Women diagnosed during the 6–12 months period post-delivery had similar proportion of triple assessment (i.e. mammography, ultrasound, biopsy) as women with non-PABC. There was no difference in fine needle aspiration (FNA) or core biopsy use among women with PABC and non-PABC.

**Table 2:** Symptoms, mode of detection, and diagnostic intensity by pregnancy-association at diagnosis

	Pregnancy and 0–24 months postpartum				Pregnancy				0–6 months				6–12 months				12–24 months			
	PABC		Non-PABC (matched)		PABC		Non-PABC (matched)		PABC		Non-PABC (matched)		PABC		Non-PABC (matched)		PABC		Non-PABC (matched)	
	*N*	%	*N*	%	*N*	%	*N*	%	*N*	%	*N*	%	*N*	%	*N*	%	*N*	%	*N*	%
Symptoms^a^																				
Lump or swelling in breast or axilla	243	96.4	239	94.8	37	97.4	37	97.4	28	100.0	27	96.4	58	96.7	57	95.0	120	95.2	118	93.7
Pain	20	8.0	30	12.0	2	5.3	4	10.5	3	11.1	4	14.8	1	1.7	6	10.0	14	11.1	16	12.7
Nipple changes	5	2.0	5	2.0	2	5.3	1	2.6	0	0.0	1	3.6	2	3.3	1	1.7	1	0.8	2	1.6
Nipple discharge	6	2.4	4	1.6	0	0.0	2	5.3	3	10.7	0	0.0	0	0.0	1	1.7	3	2.4	1	0.8
Other^b^	6	2.4	17	6.9	1	2.8	0	0.0	0	0.0	3	11.1	1	1.7	5	8.6	4	3.2	9	7.2
*p* value (lump)		0.39			1.00				0.32				0.65				0.59			
*p* value (pain)		0.12			0.41				0.71				0.06				0.67			
Number of symptoms																				
0 or missing	14	5.1	7	2.6	2	4.9	1	2.4	1	3.3	1	3.3	2	3.1	2	3.1	9	6.5	3	2.2
1	228	83.5	221	81.0	34	82.9	32	78.0	23	76.7	22	73.3	57	89.1	53	82.8	114	82.6	114	82.6
2	30	11.0	42	15.4	5	12.2	8	19.5	6	20.0	7	23.3	5	7.8	8	12.5	14	10.1	19	13.8
3	1	0.4	3	1.1	0	0.0	0	0.0	0	0.0	0	0.0	0	0.0	1	1.6	1	0.7	2	1.4
Total	273	100.0	273	100.0																
*p* value		0.14			0.61				0.96				0.64				0.25			
Suspected tumour/signs discovered by																				
Patient	223	96.1	216	93.1	33	97.1	32	94.1	27	96.4	28	100.0	57	100.0	53	93.0	106	93.8	103	91.2
Doctor, midwife, Nurse	4	1.7	8	3.4	0	0.0	1	2.9	1	3.6	0	0.0	0	0.0	2	3.5	3	2.7	5	4.4
Patient’s partner, screening, other	5	2.2	8	3.4	1	2.9	1	2.9	0	0.0	0	0.0	0	0.0	2	3.5	4	3.5	5	4.4
Total	232	100.0	232	100.0																
*p* value		0.37			0.61				0.32				0.14				0.71			
First point of contact with healthcare																				
Primary care doctor, midwife, nurse	139	61.0	141	61.8	18	56.3	20	62.5	13	48.1	16	59.3	34	65.4	35	67.3	74	63.2	70	59.8
Specialist	88	38.6	84	36.8	14	43.8	12	37.5	14	51.9	10	37.0	18	34.6	17	32.7	42	35.9	45	38.5
Other/screening	1	0.4	3	1.3	0	0.0	0	0.0	0	0.0	1	3.7	0	0.0	0	0.0	1	0.9	2	1.7
Total	228	100.0	228	100.0																
*p* value		0.59			0.62				0.47				0.86				0.78			
Triple assessment^c^																				
Mammo/US/biopsy	197	77.3	211	82.7	22	56.4	27	69.2	21	75.0	24	85.7	47	77.0	50	82.0	107	84.3	110	86.6
Mammo/US	3	1.2	4	1.6	1	2.6	1	2.6	0	0.0	0	0.0	0	0.0	0	0.0	2	1.6	3	2.4
Mammo/biopsy	29	11.4	30	11.8	4	10.3	8	20.5	2	7.1	3	10.7	8	13.1	8	13.1	15	11.8	11	8.7
US/biopsy	16	6.3	3	1.2	7	17.9	2	5.1	4	14.3	0	0.0	4	6.6	1	1.6	1	0.8	0	0.0
Mammography only	1	0.4	0	0.0	0	0.0	0	0.0	0	0.0	0	0.0	0	0.0	0	0.0	1	0.8	0	0.0
US only	0	0.0	2	0.8	0	0.0	1	2.6	0	0.0	1	3.6	0	0.0	0	0.0	0	0.0	0	0.0
Biopsy only	9	3.5	5	2.0	5	12.8	0	0.0	1	3.6	0	0.0	2	3.3	2	3.3	1	0.8	3	2.4
Total	255	100.0	255	100.0																
*p* value		0.05			0.09				0.14				0.61				0.54			
Mammography																				
No	25	9.8	10	3.9	12	30.8	3	7.7	5	17.9	1	3.6	6	9.8	3	4.9	2	1.6	3	2.4
Yes	230	90.2	245	96.1	27	69.2	36	92.3	23	82.1	27	96.4	55	90.2	58	95.1	125	98.4	124	97.6
Total	255	100.0	255	100.0																
*p* value		< 0.01			0.01				0.10				0.32				0.65			
Ultrasound (US)																				
No	10	4.8	5	2.4	3	10.3	1	3.4	2	7.7	0	0.0	3	6.3	1	2.1	2	1.9	3	2.8
Yes	200	95.2	205	97.6	26	89.7	28	96.6	24	92.3	26	100.0	45	93.8	47	97.9	105	98.1	104	97.2
Total	210	100.0	210	100.0																
*p* value		0.10			0.16				0.16				0.16				0.56			
Biopsy^d^																				
No	1	0.4	1	0.4	0	0.0	0	0.0	1	3.6	0	0.0	0	0.0	0	0.0	0	0.0	1	0.8
FNA only	219	88.7	206	83.4	34	94.4	32	88.9	21	75.0	22	78.6	56	91.8	53	86.9	108	88.5	99	81.1
Core biopsy w/wo FNA	27	10.9	40	16.2	2	5.6	4	11.1	6	21.4	6	21.4	5	8.2	8	13.1	14	11.5	22	18.0
Total	247	100.0	247	100.0																
*p* value		0.19			0.32				0.61				0.41				0.16			

*p* values from paired Stuart-Maxwell test (Mc Nemar’s test) including only matched pairs with complete information

*FNA* Fine-needle aspiration, *mammo* mammography, *US* Ultrasound

^a^Multiple symptoms can be reported, thus percentages might exceed 100%. Tests for symptom are binary (yes/no) for lump and pain, too few observations for testing other symptoms

^b^Other category includes skin changes, fatigue, weight loss and other

^c^Biopsy includes core biopsy and/or FNA

^d^Core biopsy includes core biopsy with/without FNA

### Surgery and adjuvant treatment

Women with PABC more often underwent mastectomy compared to matched controls (68.6% vs. 57.9% in matched controls, *p* = 0.02), a finding which was most pronounced in women diagnosed 0–6 months post-delivery (83.3% vs. 56.7%, *p* = 0.06) (Table 3). Compared to non-PABC, the rates of mastectomy were higher in women with PABC diagnosed in 1992–1997 (PABC: 74% mastectomy, non-PABC: 46%) and 1998–2003 (PABC: 63% mastectomy, non-PABC: 48%), but not 2004–2009 (PABC: 69% mastectomy, non-PABC: 63%). There was a significant difference in the use of sentinel node biopsy and axillary clearance by PABC status (sentinel node: 18.4% vs. 27.6%, clearance: 78.2% vs. 66.7%, *p* = 0.03). Axillary clearance was performed in 90% of women diagnosed within 0–6 months after delivery reflecting the higher proportion of node-positive disease. Overall, PABC as a group received RT/CT or CT alone to a higher extent and less ET compared to non-PABC, in particular in patients diagnosed during pregnancy and within 6 months of delivery. Restricting to patients with ER positive disease, women with PABC had lower rates of ET compared to matched controls (73.3% vs. 86.7%, borderline significant, *p* = 0.059).

**Table 3:** Treatment by pregnancy-association at diagnosis

	Pregnancy and 0–24 months postpartum				Pregnancy				0–6 months				6–12 months				12–24 months			
	PABC		Non-PABC (matched)		PABC		Non-PABC (matched)		PABC		Non-PABC (matched)		PABC		Non-PABC (matched)		PABC		Non-PABC (matched)	
	*N*	%	*N*	%	*N*	%	*N*	%	*N*	%	*N*	%	*N*	%	*N*	%	*N*	%	*N*	%
Surgery																				
No surgery	6	2.3	4	1.5	1	2.6	0	0.0	1	3.3	0	0.0	1	1.6	1	1.6	3	2.3	3	2.3
Mastectomy	179	68.6	151	57.9	26	66.7	24	61.5	25	83.3	17	56.7	47	75.8	41	66.1	81	62.3	69	53.1
BCS	76	29.1	106	40.6	12	30.8	15	38.5	4	13.3	13	43.3	14	22.6	20	32.3	46	35.4	58	44.6
Total	261	100.0	261	100.0																
*p* value		0.02			0.48				0.06				0.40				0.33			
Axillary surgery																				
No axillary surgery	8	3.1	12	4.6	2	5.1	3	7.7	1	3.3	0	0.0	2	3.2	2	3.2	3	2.3	7	5.4
Clearance	204	78.2	174	66.7	29	74.4	24	61.5	27	90.0	24	80.0	55	88.7	47	75.8	93	71.5	79	60.8
Sentinel node biopsy	48	18.4	72	27.6	8	20.5	11	28.2	2	6.7	6	20.0	5	8.1	12	19.4	33	25.4	43	33.1
Sampling	1	0.4	3	1.1	0	0.0	1	2.6	0	0.0	0	0.0	0	0.0	1	1.6	1	0.8	1	0.8
Total	261	100.0	261	100.0																
*p* value		0.03			0.48				0.32				0.23				0.30			
Adjuvant therapy (planned)																				
No adjuvant trt	25	9.6	10	3.8	5	13.2	5	13.2	2	6.7	0	0.0	4	6.5	0	0.0	14	10.8	5	3.8
RT/CT/ET	62	23.8	90	34.6	8	21.1	15	39.5	8	26.7	12	40.0	15	24.2	21	33.9	31	23.8	42	32.3
RT/CT	94	36.2	65	25.0	16	42.1	6	15.8	11	36.7	5	16.7	27	43.5	20	32.3	40	30.8	34	26.2
RT/ET	19	7.3	24	9.2	1	2.6	2	5.3	1	3.3	4	13.3	5	8.1	5	8.1	12	9.2	13	10.0
CT/ET	19	7.3	20	7.7	1	2.6	3	7.9	2	6.7	2	6.7	6	9.7	5	8.1	10	7.7	10	7.7
RT only	8	3.1	11	4.2	2	5.3	0	0.0	1	3.3	2	6.7	0	0.0	2	3.2	5	3.8	7	5.4
CT only	25	9.6	23	8.8	5	13.2	3	7.9	5	16.7	3	10.0	5	8.1	3	4.8	10	7.7	14	10.8
ET only	8	3.1	17	6.5	0	0.0	4	10.5	0	0.0	2	6.7	0	0.0	6	9.7	8	6.2	5	3.8
Total	260	100.0	260	100.0																
*p* value		< 0.01			0.08				0.31				0.07				0.20			
Radiotherapy (planned)																				
RT no	77	29.6	70	26.9	11	28.9	15	39.5	9	30.0	7	23.3	15	24.2	14	22.6	42	32.3	34	26.2
RT yes	183	70.4	190	73.1	27	71.1	23	60.5	21	70.0	23	76.7	47	75.8	48	77.4	88	67.7	96	73.8
Total	260	100.0	260	100.0	38	100.0	38	100.0	30	100.0	30	100.0	62	100.0	62	100.0	130	100.0	130	100.0
*p* value		0.51			0.32				0.56				0.84				0.29			
Chemotherapy (planned)																				
CT no	60	23.1	62	23.8	8	21.1	11	28.9	4	13.3	8	26.7	9	14.5	13	21.0	39	30.0	30	23.1
CT yes	200	76.9	198	76.2	30	78.9	27	71.1	26	86.7	22	73.3	53	85.5	49	79.0	91	70.0	100	76.9
Total	260	100.0	260	100.0	38	100.0	38	100.0	30	100.0	30	100.0	62	100.0	62	100.0	130	100.0	130	100.0
*p* value		0.83			0.41				0.25				0.35				0.18			
Endocrine treatment (planned)																				
ET no	152	58.5	109	41.9	28	73.7	14	36.8	19	63.3	10	33.3	36	58.1	25	40.3	69	53.1	60	46.2
ET yes	108	41.5	151	58.1	10	26.3	24	63.2	11	36.7	20	66.7	26	41.9	37	59.7	61	46.9	70	53.8
Total	260	100.0	260	100.0	38	100.0	38	100.0	30	100.0	30	100.0	62	100.0	62	100.0	130	100.0	130	100.0
*p* value		< 0.01			< 0.01				0.04				0.06				0.28			
Endocrine treatment in ER+ (planned)^a^																				
ET no	16	26.7	8	13.3	3	37.5	1	12.5	3	60.0	1	20.0	2	20.0	0	0.0	8	21.6	6	16.2
ET yes	44	73.3	52	86.7	5	62.5	7	87.5	2	40.0	4	80.0	8	80.0	10	100.0	29	78.4	31	83.8
Total	60	100.0	60	100.0	8	100.0	8	100.0	5	100.0	5	100.0	10	100.0	10	100.0	37	100.0	37	100.0
*p* value		0.059				0.16				0.32				0.16				0.53		
Oophorectomy (planned)																				
No	257	97.7	250	95.1	34	97.1	35	100.0	28	96.6	29	100.0	60	100.0	56	93.3	124	96.9	119	93.0
Yes	6	2.3	13	4.9	1	2.9	0	0.0	1	3.4	0	0.0	0	0.0	4	6.7	4	3.1	9	7.0
Total	10		10																	
*p* value		0.11			0.32				0.32				0.05				0.17			

*p* values from paired Stuart–Maxwell test (Mc Nemar’s test) including only matched pairs with complete information

^a^Among matched pairs where both PABC/non-PABC were ER+ (*n* = 120)

### Timing of symptoms, diagnosis and treatment

In women diagnosed with breast cancer during pregnancy, nearly all experienced first symptoms during pregnancy and more than half also had surgery during pregnancy (Supplemental Table 2). For women diagnosed during 0–6 months post-delivery, about half reported initial symptoms during pregnancy. Very few of the women diagnosed 6–24 months after delivery had initial symptoms during pregnancy.

### Time from initial symptoms to first contact with healthcare (A)

**Fig. 1:**
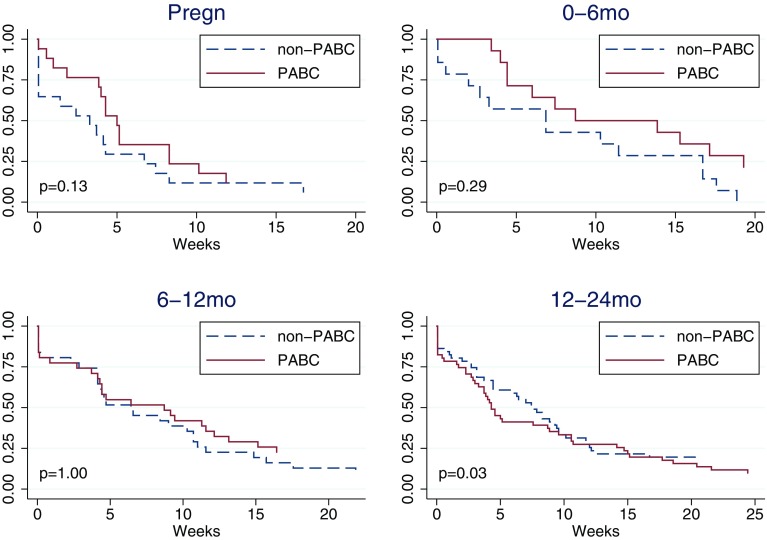
Kaplan–Meier curve of time from first symptom to first healthcare contact (A) by pregnancy-association at diagnosis. Controls (non-PABC) includes those age- and hospital-matched to PABC in each specific PABC window. *p* values from stratified log-rank test including only matched pairs with complete information. Based on *N* = 113 matched pairs PABC/non-PABC, of which during pregnancy 17 pairs, 0–6 months 14 pairs, 6–12 months 31 pairs, 12–24 months 51 pairs

Women diagnosed during pregnancy had a median of 35 days from symptoms to first contact with healthcare, which was non-significantly longer than for matched controls (median 23 days) (Table 4; Fig. 1). For women diagnosed 0–6 and 6–12 months following delivery, the corresponding waiting times were 61 days in both groups (median 61 vs. 48 and 61 vs. 45, respectively), while the waiting time in women diagnosed 12–24 months post-delivery was shorter compared to matched controls (median 30 vs. 52 days, *p* = 0.03).

**Table 4:** Times from symptoms to diagnosis to treatment by pregnancy-association at diagnosis

	Pregnancy		0–6 months		6–12 months		12–24 months	
	PABC	Non-PABC (matched)	PABC	Non-PABC (matched)	PABC	Non-PABC (matched)	PABC	Non-PABC (matched)
	*N* (%)	*N* (%)	*N* (%)	*N* (%)	*N* (%)	*N* (%)	*N* (%)	*N* (%)
Time from symptoms to healthcare contact (A) in days								
0–30	6 (35.3)	11 (64.7)	2 (14.3)	6 (42.9)	10 (32.3)	11 (35.5)	24 (47.1)	17 (33.3)
31–60	7 (41.2)	4 (23.5)	4 (28.6)	2 (14.3)	6 (19.4)	7 (22.6)	7 (13.7)	12 (23.5)
61–180	2 (11.8)	1 (5.9)	5 (35.7)	6 (42.9)	9 (29)	10 (32.3)	15 (29.4)	13 (25.5)
> 180	2 (11.8)	1 (5.9)	3 (21.4)	0 (0)	7 (22.6)	3 (9.7)	5 (9.8)	9 (17.6)
Total	17 (100)	17 (100)	14 (100)	14 (100)	31 (100)	31 (100)	51 (100)	51 (100)
Median days	35	23	61	48	61	45	30	52
*p* value	0.13		0.29		1.00		0.03	
Time from healthcare contact to diagnosis (B) in days								
0–15	27 (79.4)	22 (64.7)	22 (78.6)	15 (53.6)	34 (61.8)	36 (65.5)	74 (63.8)	76 (65.5)
16–30	4 (11.8)	6 (17.6)	3 (10.7)	6 (21.4)	15 (27.3)	11 (20)	25 (21.6)	20 (17.2)
31–60	2 (5.9)	4 (11.8)	3 (10.7)	4 (14.3)	2 (3.6)	7 (12.7)	12 (10.3)	13 (11.2)
> 60	1 (2.9)	2 (5.9)	0 (0)	3 (10.7)	4 (7.3)	1 (1.8)	5 (4.3)	7 (6)
Total	34	34	28	28	55	55	116	116
Median days	2	6	1	10	9	9	8	5
p value	0.86		0.11		0.41		0.36	
Time from diagnosis to treatment (C), in days								
0–15	6 (17.6)	4 (11.8)	10 (35.7)	4 (14.3)	19 (34.5)	8 (14.5)	34 (29.3)	19 (16.4)
16–30	17 (50)	15 (44.1)	11 (39.3)	15 (53.6)	27 (49.1)	22 (40)	41 (35.3)	56 (48.3)
31–60	10 (29.4)	14 (41.2)	5 (17.9)	8 (28.6)	7 (12.7)	22 (40)	33 (28.4)	37 (31.9)
> 60	1 (2.9)	1 (2.9)	2 (7.1)	1 (3.6)	2 (3.6)	3 (5.5)	8 (6.9)	4 (3.4)
Total	34	34	28	28	55	55	116	116
Median days	21	27	19	26	19	27	24	25
*p* value	0.09		0.45		< 0.01		0.23	
Time from healthcare contact to treatment (D) in days								
0–15	2 (5.9)	0 (0)	5 (17.9)	1 (3.6)	5 (9.1)	1 (1.8)	9 (7.8)	6 (5.2)
16–30	12 (35.3)	13 (38.2)	14 (50)	9 (32.1)	21 (38.2)	19 (34.5)	33 (28.4)	37 (31.9)
31–60	15 (44.1)	13 (38.2)	6 (21.4)	11 (39.3)	23 (41.8)	24 (43.6)	49 (42.2)	52 (44.8)
> 60	5 (14.7)	8 (23.5)	3 (10.7)	7 (25)	6 (10.9)	11 (20)	25 (21.6)	21 (18.1)
Total	34	34	28	28	55	55	116	116
Median days	30	40	26	37	32	35	39	37
*p* value	0.22		< 0.01		0.08		0.35	

*p* value from stratified logrank test (stratified on matching) including only matched pairs with complete information

### Time from first healthcare contact to diagnosis (B)

**Fig. 2:**
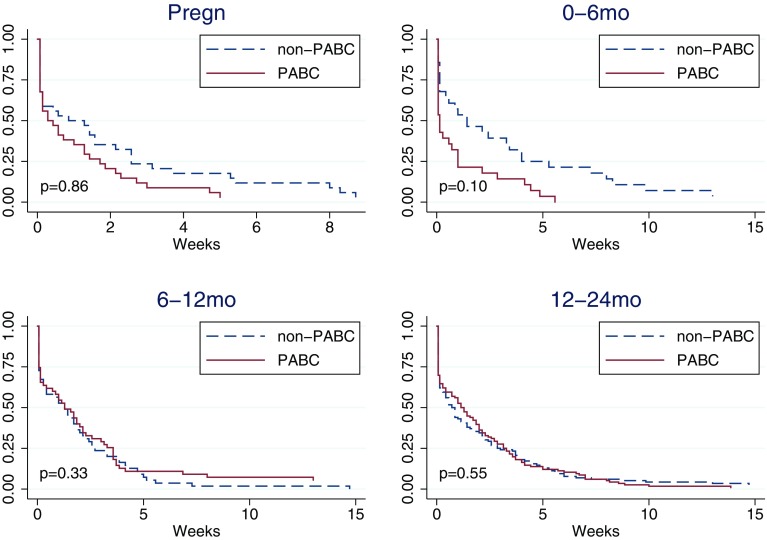
Kaplan–Meier curve of time from first healthcare contact to diagnosis (B) by pregnancy-association at diagnosis. Controls (non-PABC) includes those age- and hospital-matched to PABC in each specific PABC window. *p* values from stratified log-rank test including only matched pairs with complete information. Based on *N* = 233 matched pairs PABC/non-PABC, of which during pregnancy 34 pairs, 0–6 months 28 pairs, 6–12 months 55 pairs, 12–24 months 116 pairs

Median waiting time from first healthcare contact to diagnosis was 2 days (vs. 6 days in controls) for women diagnosed during pregnancy, and 1 day (vs. 10 days in controls) for women diagnosed 0–6 months after delivery (Table 4; Fig. 2). Median waiting times in women diagnosed 6–12 or 12–24 months were 9 and 8 days, respectively, which were similar to matched controls.

### Time from diagnosis to start of treatment (C)

**Fig. 3:**
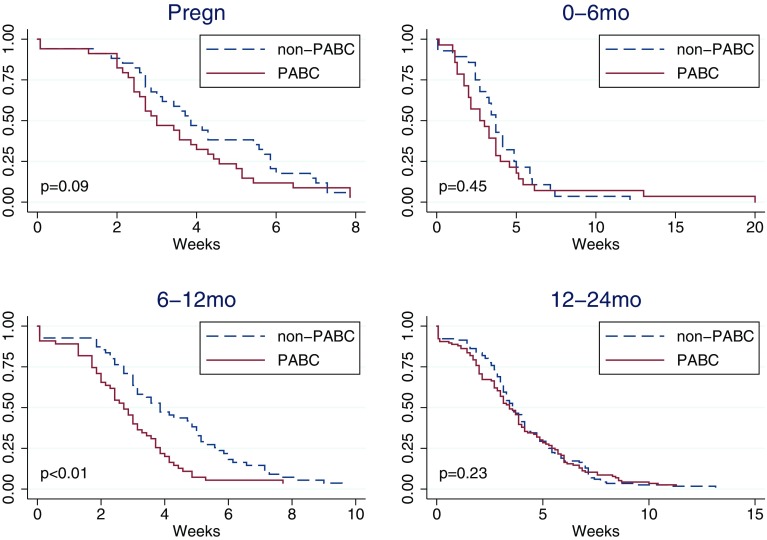
Kaplan–Meier curve of time from diagnosis to treatment initiation (C) by pregnancy-association at diagnosis. Controls (non-PABC) includes those age- and hospital-matched to PABC in each specific PABC window. *p* values from stratified log-rank test including only matched pairs with complete information. Based on *N* = 233 matched pairs PABC/non-PABC, of which during pregnancy 34 pairs, 0–6 months 28 pairs, 6–12 months 55 pairs, 12–24 months 116 pairs

Women diagnosed during pregnancy had shorter waiting time from diagnosis to treatment initiation compared to matched controls (median 21 vs. 27 days, non-significant), as had women diagnosed 0–6 (19 vs. 26 days, non-significant) and 6–12 months after delivery (19 vs. 27 days, significant) (Table 4; Fig. 3). From the medical records, there was evidence of planned postponed treatment in 5 out of 38 women diagnosed during pregnancy.

### Time from first healthcare contact to start of treatment (D)

**Fig. 4:**
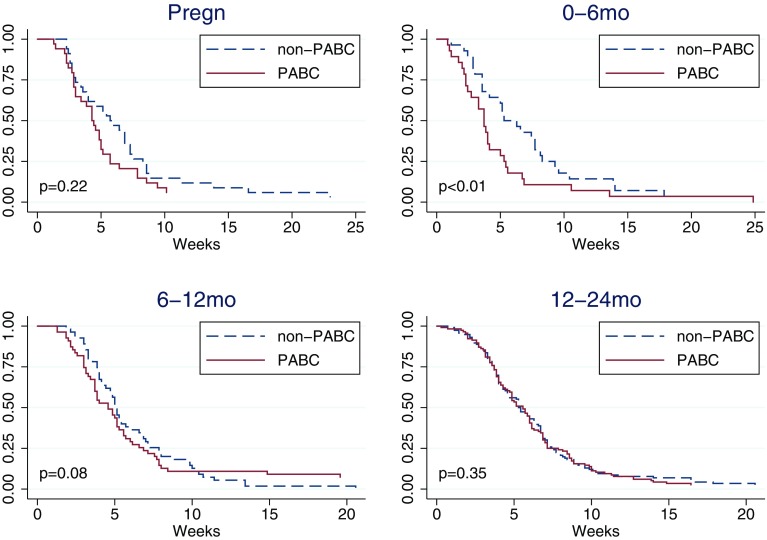
Kaplan–Meier curve of time from healthcare contact to treatment initiation (D) by pregnancy-association at diagnosis. Controls (non-PABC) includes those age- and hospital-matched to PABC in each specific PABC window. *p* values from stratified log-rank test including only matched pairs with complete information. Based on *N* = 233 matched pairs PABC/non-PABC, of which during pregnancy 34 pairs, 0–6 months 28 pairs, 6–12 months 55 pairs, 12–24 months 116 pairs

Overall, women diagnosed during pregnancy or 0–6 months after delivery had the shortest time from first contact with healthcare to start of treatment. This difference was significant (*p* < 0.01) in women diagnosed 0–6 months after delivery compared to control women (Table 4; Fig. 4). Median waiting times from first healthcare contact to treatment initiation were 30, 26, 32, and 39 days, for women diagnosed during pregnancy, 0–6 months, 6–12 months, and 12–24 months following delivery, respectively.

## Discussion

We found no evidence of delayed diagnosis or treatment in women with PABC following a first healthcare contact. If anything, waiting times in all steps from initial investigation to start of treatment were shorter in women with PABC. However, there was an indication of longer time between symptoms to first healthcare contact in women with PABC during pregnancy and early lactation.

Compared to matched control women with breast cancer, women with PABC had similar symptoms but more often underwent biopsy and ultrasound than mammography during initial work-up. Women with PABC had shorter waiting time to initiation of treatment compared to non-PABC (non-significant) and less often received ET. However, when we also accounted for ER status, women with PABC still had lower rates of ET, indicating that tumour biology alone cannot fully explain the lower rates of ET in these patients. With respect to surgery, mastectomy and axillary clearance were more often performed in women diagnosed 0–12 months after delivery, reflecting mainly a more advanced stage of disease at diagnosis rather than the association with pregnancy.

Our results do not support the hypothesis of diagnostic or treatment delays in women with PABC. Furthermore, patterns of management in women diagnosed 12–24 months after delivery were similar to that in non-PABC women, indicating a transient effect of pregnancy-association on detection, diagnostic procedures and treatment.

Results from several previous studies suggest the presence of diagnostic delays in women with PABC both on the part of the patient and the healthcare provider [9, 15, 16, 19, 20, 24]. However, one study did not find evidence for diagnostic delays [18]. One French study, including 22 pregnant women and 80 women with breast cancer 1 year post-partum, found a mean diagnostic delay of 5.8 months [19], which is considerably longer than in the present study. It cannot be excluded that changes in public and clinical awareness over calendar time could contribute to differences between findings in historical and more recent studies. Most studies have included time from symptoms to diagnosis as ‘diagnostic delay’, while our findings suggest that time from symptoms to healthcare contact may be delayed but not time from healthcare contact to diagnosis. Signs and symptoms being misinterpreted as pregnancy-related are likely to contribute to delays since most breast symptoms during pregnancy are not due to breast cancer. A majority of studies have found that the main symptom in women with PABC is a palpable mass, which is similar to other young women with breast cancer [15, 16, 19, 20].

Because of the lower sensitivity of mammography in pregnant and lactating women [8, 9, 25], our finding of a lower use of mammography in PABC women was expected during the period under study. Today digital mammography will be used also on pregnant patients, in addition to the recommended ultrasound and biopsies [8, 11]. Core biopsy is recommended for all patients, despite the risk for bleeding and fistula complications, since FNA has a lower sensitivity in pregnant and lactating women [6–8]. Corroborating results from an earlier study [20], our findings suggest no difference in the type of biopsy used in women with PABC.

According to recent management guidelines, women with PABC should receive treatment similar to other young BC patients, i.e. based on stage and tumour biology, with the exception of endocrine therapy, anti-HER2 therapy and radiotherapy, which are contraindicated in pregnant women and need to be postponed until after delivery. The observed higher rate of mastectomy in women with PABC were most likely due to more advanced stage at diagnosis among these patients. Another explanation is calendar time effects, since patients with PABC were more often diagnosed in the earlier period than patients with non-PABC. Compared to non-PABC, the rates of mastectomy were higher in women diagnosed with PABC before year 2000. A third explanation for the higher rates of mastectomy among PABC is that neoadjuvant chemotherapy to pregnant patients was not yet established in clinical practice in Sweden during the study period. Primary surgery in early pregnancy excluded BCS as an option as radiotherapy is contraindicated during pregnancy. In addition, women with PABC more often had HER2 positive tumours, which is associated with higher rates of multifocality and subsequent mastectomy. However, there was no difference in rates of multifocality between PABC and non-PABC patients in our material. Previous studies have found similar rates of mastectomy and axillary clearance in women with PABC [26–29]. However, one large population-based study found higher rates of mastectomy in women with PABC both during pregnancy and post-partum [30]. Most earlier studies have also found similar use of chemotherapy and radiotherapy, and a lower use of endocrine therapy in women with PABC [27–31], although a large study reported more use of chemotherapy in pregnant women than in matched controls [26]. Similar to our study, only three studies have been able to separate pregnant from postpartum cases when assessing treatment patterns in comparison to non-PABC [26, 29, 30]. With diverging results, only a few smaller studies have assessed treatment delays in women with PABC [21, 24, 29].

Strengths of our study included the relatively large size, the population-based setting, a virtually complete register based ascertainment of PABC and non-PABC status, and the extraction of chart data by trained nurses based on a standardised protocol. The uniform and tax funded Swedish healthcare system reduced the likelihood of large differences in management across centers. In addition, the matched design accounted for any remaining differences between hospitals. The matched design also minimised the potential confounding by age between PABC cases and non-PABC controls. Furthermore, we were able to separate the PABC window into pregnancy and postpartum periods reflecting differences in tumour biology, mammographic density and breastfeeding prevalence. During 0–6 months postpartum, most Swedish women breastfeed, and we have previously reported adverse tumour characteristics (i.e. triple-negative, nodal spread) and a poorer prognosis in this patient group [5, 32, 33]. During 6–12 months postpartum, breastfeeding occurs partially, while only a few women still breastfeed between months 12–24 months postpartum. However, an improved prognosis has been observed in both these time windows (6–12, 12–24 months postpartum) compared to the pregnancy and 0–6 months postpartum windows [5, 32]. Hence, it is of particular importance to evaluate delays in diagnosis and management in the pregnancy and early postpartum windows separately. Since pregnancy-induced physiological changes may mask a developing breast tumour, our study focused on the 2 year period after delivery where delays in diagnosis and management are most likely to be directly pregnancy-related.

Several limitations need mentioning. Some of the data extracted from the medical records were incomplete, in particular on timing of signs and symptoms. The nurses abstracting data were not blinded for case status of the patient, and no extraction was performed by both nurses from the same charts. Also, it cannot be excluded that results may have been influenced by the selection of hospitals. All the included PABC patients were diagnosed and treated at large centers, and may not be fully representative for patients treated at smaller hospitals. However, an absolute majority of women with PABC in Sweden are referred to larger hospitals for specialised multi-disciplinary assessment and treatment. Furthermore, the study was not matched on calendar period and differences reflecting changing practices over time cannot be ruled out. However, the study period was fairly short (1992–2009), with most women diagnosed in the 2000s, and a sensitivity analysis accounting for period confirmed the results. Another potential limitation is the generalisability of the findings to other settings and populations. Since long, Sweden has had national management guidelines for breast cancer with high rates of adherence. Regional care guidelines have included recommendations for PABC for the period under study. Another limitation of our study was the absence of detailed information on given adjuvant systemic treatment, including type of chemotherapy regimens and use of targeted therapies. While our study focused on the immediate period after delivery, future studies need also assess the possible diagnostic and management delays in women diagnosed further away from delivery.

In conclusion, the present results do not support the notion of healthcare provider diagnostic and treatment delays in women with PABC. However, our findings indicate that the patient´s own initial healthcare contact may be delayed in pregnant and lactating women. Women with PABC receive similar diagnostic workup, with consideration given to pregnancy, and treatment reflecting stage and tumour biology. The previously observed poorer prognosis in patients with PABC in this study population is therefore unlikely to reflect diagnostic or treatment delays [5, 32, 33].

## Electronic supplementary material

Below is the link to the electronic supplementary material.

Supplementary material 1 (PDF 364 KB)
