# Supplementary material for: Diagnostic pathways and management in women with pregnancy-associated breast cancer (PABC): no evidence of treatment delays following a first healthcare contact
Source: Breast Cancer Res Treat. 2018 Dec 14;174(2):489–503. doi: 10.1007/s10549-018-05083-x (PMC6422971; doi:10.1007/s10549-018-05083-x)

SUPPLEMENTAL MATERIAL

Supplemental Table 1. Sampling and retrieval rate of medical records in eligible women diagnosed with PABC and non-PABC 1992-2009 in 11 hospitals included.

Hospital	Eligible PABC 1992-2009	Eligible nonPABC 1992-2009	Sampled PABC	Sampled nonPABC (matched)	Retrieval rate[1] of PABC
	N	N	N	N	%
Retrieved from register data (n=9,441)	**778**	**8,663**			
Retrieval of medical records:					
**Non-included hospitals**	**442**	**5,602**			
**Included hospitals**	**336**	**3,061**	**273**	**273**	**81.3**
Capio S:t Görans Hospital (Stockholm)	43	443	4	4	9.3
Stockholm South General Hospital (Stockholm)	48	330	38	38	79.2
Karolinska University Hospital (Stockholm)	34	376	33	33	97.1
Danderyd Hospital (Stockholm)	37	275	36	36	97.3
Uppsala University Hospital (Uppsala)	20	186	20	20	100.0
Linköping University Hospital (Linköping)	22	177	20	20	90.9
Kristianstad Central Hospital (Kristianstad)	13	130	11	11	84.6
Skåne University Hospital (Malmö)	24	241	24	24	100.0
Skåne University Hospital (Lund)	21	273	20	20	95.2
Sahlgrenska University Hospital (Göteborg)	37	364	33	33	89.2
Sahlgrenska University Hospital (Mölndal)	37	266	34	34	91.9

[1] Retrieval rate among PABC: 273/336=0.813. Retrieval rate=sampled PABC/eligible PABC

Supplemental Table 2. Timing of symptoms, contact, examination and surgery in relation to during pregnancy or during lactation by pregnancy-association at diagnosis.

Timing of diagnosis	Pregnancy and 0-24 mo postpartum		Pregnancy	0-6mo	6-12mo	12-24mo
	PABC	Non-PABC (matched)	PABC	PABC	PABC	PABC
	N	N	N	N	N	N
**Timing of symptoms**						
Non-PABC	0	229	0	0	0	0
Symptoms during pregnancy	61	2	38	13	7	3
Symptoms during lactation	50	0	0	14	26	10
Symptoms after lactation	120	0	0	2	24**	94
Unknown timing of symptoms[1]	42	42	3	1	7	31
**Timing of contact**						
Non-PABC	0	234	0	0	0	0
Contact during pregnancy	47	2	40	2	3	2
Contact during lactation	55	0	0	25	24	6
Contact after lactation	134	0	0	3	32	99
Unknown timing of contact[1]	37	37	1	0	5	31
**Timing of examination**						
Non-PABC	0	228	0	0	0	0
Examination during pregnancy	45	1	40	1	1	2
Examination during lactation	50	0	0	23	22	5
Examination after lactation	135	0	0	5	36	94
Unknown timing of examination[1]	88	44	1	1	5	37
**Timing of surgery**						
Non-PABC	0	214	0	0	0	0
Surgery during pregnancy	26	1	24	0	0	1
Surgery during lactation	13	0	2	4	7	0
Surgery after lactation	176	0	9	21	46	100
Unknown timing of surgery[1]	117	58	6	5	11	37

[1] Unknown includes PABC with unknown timing, and their matched non-PABC controls.

Supplemental Figure 1. Definition of waiting times (A, B, C, D)

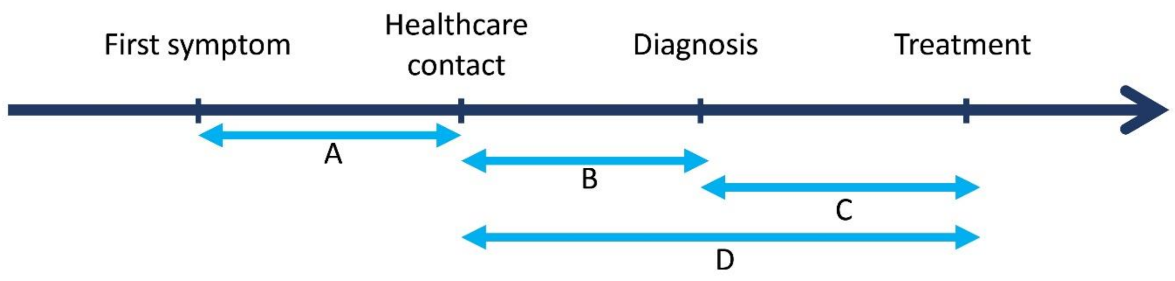

Supplement: Supplementary file 1 — Supplementary material 1 (PDF 364 KB) [file 10549_2018_5083_MOESM1_ESM.pdf]
